# Genetic and Clinical Findings in an Ethnically Diverse Cohort with Retinitis Pigmentosa Associated with Pathogenic Variants in CERKL

**DOI:** 10.3390/genes11121497

**Published:** 2020-12-12

**Authors:** Susan M. Downes, Tham Nguyen, Vicky Tai, Suzanne Broadgate, Mital Shah, Saoud Al-Khuzaei, Robert E. MacLaren, Morag Shanks, Penny Clouston, Stephanie Halford

**Affiliations:** 1 Oxford Eye Hospital, John Radcliffe Hospital, Oxford University Hospitals NHS Foundation Trust, Oxford OX3 9DU, UK; Tham.Nguyen@ouh.nhs.uk (T.N.); tai.vicky18@gmail.com (V.T.); mital.shah@nhs.net (M.S.); saoud.al-khuzaei@stx.ox.ac.uk (S.A.-K.); enquiries@eye.ox.ac.uk (R.E.M.); 2 Nuffield Laboratory of Ophthalmology, Nuffield Department of Clinical Neuroscience, University of Oxford, Level 6 John Radcliffe Hospital, Headley Way, Oxford OX3 9DU, UK; suzanne.broadgate@ndcn.ox.ac.uk; 3 Oxford Medical Genetics Laboratories, Oxford University Hospitals NHS Foundation Trust, Oxford OX3 7LE, UK; Morag.Shanks@ouh.nhs.uk (M.S.); Penny.Clouston@ouh.nhs.uk (P.C.)

**Keywords:** *CERKL*, inherited retinal dystrophy (IRD), retinitis pigmentosa (RP), autosomal recessive (ar), variable phenotype

## Abstract

Autosomal recessive retinitis pigmentosa is caused by mutations in over 40 genes, one of which is the ceramide kinase-like gene (*CERKL*). We present a case series of six patients from six unrelated families diagnosed with inherited retinal dystrophies (IRD) and with two variants in *CERKL* recruited from a multi-ethnic British population. A retrospective review of clinical data in these patients was performed and included colour fundus photography, fundus autofluorescence (AF) imaging, spectral domain–optical coherence tomography (SD–OCT), visual fields and electroretinogram (ERG) assessment where available. Three female and three male patients were included. Age at onset ranged from 7 years old to 45 years, with three presenting in their 20s and two presenting in their 40s. All but one had central visual loss as one of their main presenting symptoms. Four patients had features of retinitis pigmentosa with significant variation in severity and extent of disease, and two patients had no pigment deposition with only macular involvement clinically. Seven variants in *CERKL* were identified, of which three are novel. The inherited retinopathies associated with the *CERKL* gene vary in age at presentation and in degree of severity, but generally are characterised by a central visual impairment early on.

## 1. Introduction

Inherited retinal dystrophies (IRD) are a heterogeneous group of disorders associated with the dysfunction or death of photoreceptors, resulting in varying severity of visual loss. IRD has an incidence of 1 in 2000–3000, affecting an estimated two million people worldwide [1]. Retinitis pigmentosa (RP; Mendelian Inheritance in Man (MIM) #268000) is the most common IRD, affecting approximately 1 in 4000 people [2], characterised by symptoms of nyctalopia, peripheral visual field loss with a clinical appearance of intraretinal bone spicule pigmentation, pale discs and attenuated vasculature. RP is associated with significant genotypic and phenotypic heterogeneity, with 41 genes described in association with the autosomal recessive form to date (RetNet: https://sph.uth.edu/retnet/accessed 1 July 2020). In 1998, an RP locus (RP26) was mapped to an 11 cM region on chromosome 2q31–32 in a consanguineous Spanish family by Bayes and colleagues [3]. Subsequently, Tuson and colleagues identified a novel gene from the region that was expressed in the retina, named ceramide kinase-like (*CERKL*) [4]. The coding exons of *CERKL* were sequenced in the original RP26 family, and all patients were found to be homozygous for a nonsense mutation c.769C > T, p.Arg257* (now annotated as c.847C > T, p.Arg283*, using the numbering based on transcript NM_001030311, which consists of 14 exons) [4]. A further unrelated Spanish family was also identified with the same homozygous change [4].

CERKL shares 29% identity (50% similarity) with the ceramide kinase (CERK) protein, which phosphorylates ceramide to ceramide 1-phosphate, a sphingolipid metabolite which is involved in proliferation, apoptosis, phagocytosis and inflammation [5]. As yet, no kinase activity is reported for CERKL and its function remains unclear [6,7].

**Figure 1:**
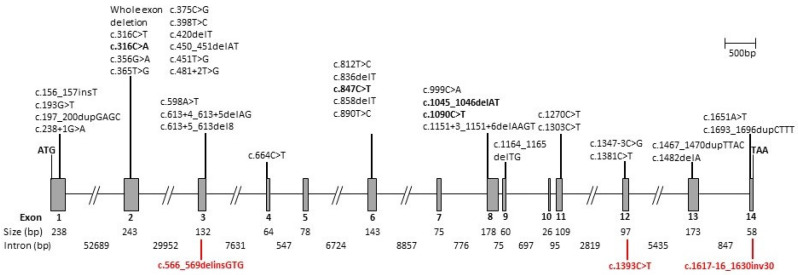
Schematic diagram of the *CERKL* genomic locus and location of identified mutations.

The *CERKL* gene is composed of 14 exons but alternative splicing produces multiple transcripts (Figure 1) [8,9]. Most of the previous studies examining variants in *CERKL* used isoform NM_201548, but this only contains 13 exons and is missing exon 5. Table 1 lists all the previously published mutations and the novel changes described in the patients in the present study, using NM_001030311 (which consists of 14 coding exons) as the reference. To date, 39 different mutations in *CERKL* have been identified (see Figure 1 and Table 1 for summary). The majority of these CERKL-associated IRD studies are case reports and many include the results from next-generation sequencing (NGS) testing with minimal phenotype data. There are, however, four studies reporting on at least four families, but these pertain to one ethnicity only in each study (Yemenite Jewish, Spanish, Tunisian and Finnish) [10,11,12,13]. In these reported populations, CERKL is a significant gene contributing to autosomal recessive RP; presumably due to a founder mutation effect.

The development of next-generation sequencing (NGS) techniques over the past 20 years led to diagnostic laboratories having the capability of screening known RP genes simultaneously [14]. Although this increased the detection rate, enabling a comprehensive genotype/phenotype analysis of cohorts of patients with same gene variants, it also identifies variants in other genes whose relevance may be difficult to determine. We report phenotypic and genotypic characterisation of six patients from six unrelated families with different ethnicities, and report three novel and four previously described variants in *CERKL*.

The *CERKL* gene on 2q31–32 consists of 14 exons and spans approximately 12 kb of genomic DNA. Exons are shown as boxes and introns as lines; all are to scale. The position of all the *CERKL* variants reported to date are marked. The mutations identified in our cohort are marked, those novel to this study are shown below in red and those reported previously are highlighted in bold above the structure (see Table 1 for details).

## 2. Materials and Methods 

The clinical notes of patients seen at the Oxford Eye Hospital, identified to have *CERKL* variants by the Oxford Medical Genetics Laboratory, were reviewed to obtain full ophthalmic details, history of symptom onset and family pedigrees. The study adhered to the tenets of the Declaration of Helsinki and was approved by the Central Oxford Research Ethics Committee and the Research and Development Department of the Oxford Radcliffe Hospitals NHS Trust (RetGene 08/H0302/96). Informed consent was obtained from all participants.

### 2.1. Clinical Studies

Clinical data collected included best corrected visual acuity, slit-lamp biomicroscopy, digital colour fundus photography, short-wavelength (488) fundus autofluorescence (Spectralis; Heidelberg Engineering, Heidelberg, Germany), spectral domain–optical coherence tomography (SD–OCT; Spectralis, Heidelberg Engineering, Heidelberg, Germany), Goldmann visual field analysis and electrodiagnostic testing, where possible. The Heidelberg software was used for retinal segmentation of the ganglion cell layer (GC) and the inner plexiform layer (IPL), which were then used to measure the thickness of the GC–IPL layers in the areas temporal and nasal to the area of macular atrophy.

### 2.2. CERKL Mutation Screening

*CERKL* variants were identified by targeted sequencing performed by the Oxford Medical Genetics Laboratory using their next generation sequencing (NGS) phenotype panels. Enrichment for the *CERKL* gene was achieved as part of a customised HaloPlex enrichment system kit (Agilent Technologies, Santa Clara, USA) designed to capture the coding exons and at least 10 bp of the flanking introns of 111 retinal genes in the retinal dystrophy panel [14]. HaloPlex reactions were prepared as per the manufacturer’s instructions. Libraries were pooled into batches of 14 and sequenced on an Illumina MiSeq instrument (Illumina) using a MiSeq v3 kit (Illumina, San Diego, USA), as per the manufacturer’s instructions. Reads were aligned using Burrow-Wheeler Aligner BWA [15] and variants were called using Platypus [49]. All variants identified by NGS were confirmed by Sanger sequencing and investigated according to the Association of Clinical Genetic Science guidelines [50]. This included filtering of candidate variants by minor allele frequency against the ExAC dataset [51].

Chromosome 2 position was based on build GRCh37/hg19 and nucleotide and protein numbering is based on *CERKL* transcript NM_001030311, comprising 14 exons. The Genome Aggregation Database (gnomAD) (available online: http://gnomad.broadinstitute.org, accessed on 1 July 2020) was used to determine the carrier frequency of the minor allele in the general population. In silico analysis using 3 different prediction methods to determine the deleteriousness of the variants, Polyphen2 [52] Sorting Intolerant from Tolerance (SIFT) [53] and Mutation Taster [54] was carried out on the variants identified. Variants in other genes in these patients identified by this methodology were also reported, and family segregation studies were performed where possible.

## 3. Results

### 3.1. Clinical Analysis

**Figure 2:**
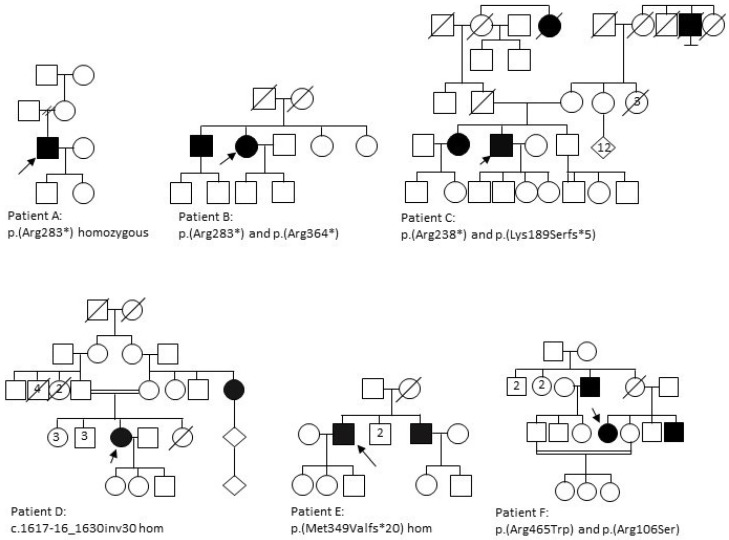
Pedigrees of families. Arrows indicate the proband and filled circles and squares represent affected females and males respectively.

**Figure 3:**
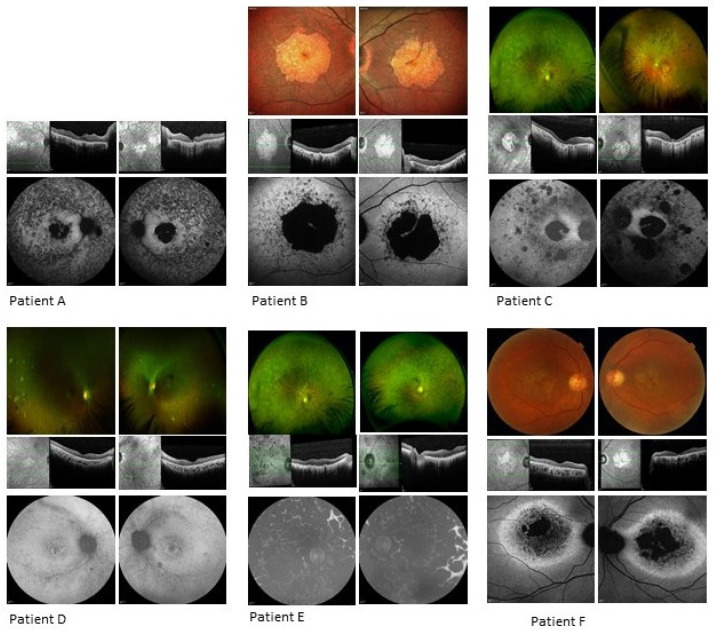
Retinal imaging of patients A to F. Each panel shows colour imaging, spectral domain–optical coherence tomography (SD–OCT) and fundus autofluorescence (FAF), showing the range of phenotypes. See Table 2 and Table 3 for further details. The thickness of the ganglion cell–inner plexiform layer (GC–IPL) layers in the areas temporal and nasal to the area of macular atrophy were also measured (see Table S1).

The clinical data of six patients from six unrelated families (Figure 2) with a diagnosis of a retinal dystrophy and with at least two variants in *CERKL* were reviewed (Figure 3, Table 2 and Table 3). The study comprised three females and three males, with age at presentation ranging from childhood to mid-40s. Symptom type and onset and clinical phenotypes are summarised in Table 2. Autofluorescence (AF) and optical coherence tomography (OCT) imaging are summarised in Table 3 and seen in Figure 3.

#### 3.1.1. Patient A

A 34-year-old white Caucasian male, homozygous for c.847C > T, p.(Arg283*) in *CERKL*, first noticed visual symptoms at the age of 25, reporting nyctalopia and difficulty with colour vision. He was fit and well other than a diagnosis of Marfan Syndrome. There was no family history of eye disease. Examination revealed visual acuities of 6/24 in the right and hand movements only in the left. Fundoscopy and imaging demonstrated bilateral attenuated vessels, intraretinal bone spicule pigmentation and patches of peripheral atrophy with central macular atrophy. AF imaging revealed significant central atrophy with foveal sparing. Patchy AF signal was noted in the mid- to far-periphery. OCT imaging showed significant central atrophy bilaterally (Figure 3, Table 2 and Table 3). Visual field testing and electrophysiology testing were not available. 

#### 3.1.2. Patient B

A 58-year-old Indian female, compound heterozygous for c.847C > T, p.(Arg283*) and c.1090C > T, p.(Arg364*) *CERKL* mutations, reported reduction of vision in both eyes at the age of 43 years. Her brother had similar symptoms at age 16, with poor central and peripheral vision. Her general health was significant for a history of iron-deficiency anaemia. Examination revealed visual acuities of 2/60 in the right and 6/36 in the left. Fundoscopy and imaging showed bilateral macular atrophy with preserved central islands. AF imaging demonstrated a central atrophic area in both maculae with central sparing and patchy mixed increased and loss of AF signal extending from atrophy to arcades. OCT imaging revealed loss of outer retina bilaterally (Figure 3, Table 2 and Table 3). Goldmann visual fields showed central reduction in sensitivity extending to 20 degrees on the right and left bilaterally (see Figure S1). Electrophysiology showed extinguished PERG, reduction in a- and b-wave amplitudes on the rod ERG and reduction in b-wave amplitude for the standard bright white flash. Cone responses showed reduction in amplitudes and the 30 Hz flicker was delayed and reduced.

#### 3.1.3. Patient C

A 56-year-old white Caucasian male, compound heterozygous for c.847C > T, p.(Arg283*) and c.566_569delinsGTG, p.(Lys189Serfs*5) *CERKL* mutations, first noticed symptoms of nyctalopia and visual loss aged 25 years. Regarding family history, his maternal great uncle was said to be visually impaired but the aetiology of this is unknown. Examination revealed visual acuities of no perception of light in both eyes. Fundoscopy and imaging revealed pale discs, grossly attenuated vasculature and central macular atrophy. Widespread intraretinal bone spicule pigmentation was noted primarily in the mid-periphery, and patchy nummular atrophic and hyper pigmented patches were observed in the mid-periphery to periphery mainly in the temporal retina. AF imaging demonstrated loss-of-signal consistent with central atrophy with a strip of minimal central sparing. There were also peripheral areas of loss-of-signal consistent with the chorioretinal atrophic patches. Fundus fluorescein angiography clearly demonstrated the central and inferior retinal patches of total retinal loss (see Figure S2). OCT imaging revealed loss of outer retina bilaterally (Figure 3, Table 2 and Table 3). Visual field testing was not performed due to poor visual acuity and electrophysiology was not available. 

#### 3.1.4. Patient D

A 30-year-old Pakistani female, homozygous for a c.1617-16_1630inv30 *CERKL* mutation, had symptoms of photopsia, photophobia, nyctalopia, abnormalities in her colour vision and central and peripheral visual field loss in her mid-twenties. Her maternal aunt was also reported to have visual impairment but of unknown aetiology. Examination revealed visual acuities of 6/18 on the right and 6/12 on the left with refraction of R: −0.50/−1.50 × 30 and L: +0.50/−1.50 × 15. Fundoscopy and imaging showed pale discs, attenuated vasculature and distinct nummular RPE depigmentation in the mid-periphery with chorioretinal atrophic patches temporally. Sparse intraretinal bone spicule pigmentation was present. AF imaging demonstrated increased signal in the maculae around the foveal loss-of-signal, with patchy, punctate loss extending from and including the arcades. OCT imaging showed focal foveal disruption of ellipsoid layer and loss of the outer retinal layers in both eyes (Figure 3, Table 2 and Table 3). Goldmann visual fields showed bilateral constriction to less than 10 degrees (see Figure S1). Electrophysiology testing showed no measurable components for PERG, cone and 30 Hz ERG, rod and standard flash and maximal ERG. 

#### 3.1.5. Patient E

A 58-year-old Indian male, homozygous for a c.1045_1046delAT, p.(Met349Valfs*20) mutation in *CERKL*, noted symptoms of nyctalopia and a slow gradual deterioration in vision in his teens, with a sharp deterioration in the last five years. His brother is also affected by the same condition (see Figure 2). Examination revealed visual acuities of perception of light bilaterally. Fundoscopy and imaging demonstrated significantly attenuated vessels, pale discs, and dense intraretinal bone spicule pigmentation scattered throughout the fundus, with central atrophy. AF imaging showed complete loss-of-signal in patches throughout the fundi, indicating severe coalescing atrophy. OCT imaging revealed loss of outer retina bilaterally (Figure 3, Table 2 and Table 3). Visual field testing was not possible due to poor visual acuity. Electrophysiology testing was not available. 

#### 3.1.6. Patient F

A 57-year-old Kashmiri female, compound heterozygous for c.1393C > T, p.(Arg465Trp) and c.316C > A, p.(Arg106Ser) mutations in *CERKL*, noticed deterioration of her central vision at age 40. Her brother and uncle were noted to have visual impairment. Examination revealed visual acuities of 6/60 bilaterally. Fundoscopy and imaging demonstrated central atrophy bilaterally. AF imaging showed significant central loss of signal denoting atrophy with a thick band of increased AF extending to the disc and around the circumference of the atrophy. OCT imaging revealed significant outer retinal loss in both eyes (Figure 3, Table 2 and Table 3). Neither visual fields nor electrophysiology were available.

### 3.2. Genetic Analysis

The six patients described here all had two disease-causing variants in *CERKL* (Figure 1). Three were homozygotes and three were compound heterozygotes (Table 2). Seven variants in *CERKL* were identified in total, three of which were novel (Figure 1, and Table 1). The most common mutation in our cohort was the nonsense variant c.847C > T, p.(Arg283*) in exon 6, which was identified in three of our patients (Patient A is homozygous and Patients B and C are both heterozygous). This is also the most common *CERKL* variant reported to date (Table 1). Another previously described nonsense mutation (c.1090C > T p.(Arg364*)) was found in Patient B [22,46]. We also found two missense mutations, one of which was novel (Figure 1, Table 1). In silico analysis was performed using three different prediction methods to determine the deleteriousness of the novel variant. Two of them, SIFT [53], and Mutation Taster [54], predicted the c.1393C > T variant to be damaging and disease-causing, respectively. However, Polyphen2 [52] predicted it to be benign. The remaining variants were a previously reported deletion, a novel indel and a novel inversion. Patient C was a compound heterozygote with c.847C > T p.(Arg283*) and the novel c.566_569delinsGTG, resulting in a frameshift and premature termination of the protein (p.(Lys189Serfs*5)). Patient D and Patient E were homozygous for a novel inversion and a novel indel respectively, the inversion (c. 1617-16_1630inv30) resulting in a 30bp inversion in exon 14, causing the splice acceptor site to be lost. The novel indel in Patient E causes a frameshift in exon 8, resulting in a premature termination of the protein. The mutations published to date, including the novel variants reported in this study, are illustrated schematically in Figure 1, demonstrating no significant clustering and they are located throughout the entire gene. 

As the patients were screened using either a panel of 55 or 111 IRD genes, variants in other genes were also described in three of them (Table S2). Patient D was heterozygous for a *PDE6A* variant (c. 769C > T p.(Arg257*)) of known pathogenicity and patient E was heterozygous for an I*MPG2* variant (c. 789C > G p.(Ser263Arg)) of uncertain pathogenicity. Patient F was heterozygous for *USH2A* c.3812-3_3837dup p.(Met1280*) of presumed pathogenicity, as well as for *NRL* c.11c > T p(Pro4Leu) of uncertain pathogenicity; these are discussed in more detail below (Table S2).

## 4. Discussion

This study of six patients from different families with pathogenic variants in *CERKL* is one of the largest multi-ethnic studies describing *CERKL*-associated IRD to date. Of the seven different pathogenic variants, three are novel. The phenotype, although variable and with different ages of onset, shows significant similarity in symptom presentation, with five of the six reporting a deterioration in central vision. Three patients were affected in their twenties and two in their forties, similar to the literature. Patient E presented at the age of seven and demonstrated the most severe phenotype. However, in the Finnish cohort described by Avela et al. the early-onset patients did not always have severe phenotypes [10]. Patients A, B, C and F showed clear central atrophy with varying degrees of a residual foveal sparing. These features were seen but less clearly demarcated in patient D, where the infrared imaging showed the central atrophic changes more distinctly and could just be an earlier stage of the same phenotype. Indeed, the phenotype of rod cone dystrophy with initially preserved central vision despite earlier macular involvement was described clearly by Khan and Abu-Safieh, who reported an almost identical phenotype [44]. Also, the peripheral nummular chorioretinal atrophic areas seen in patient D were described by Fernandez et al. in their seven patients with the c.769C > T p.(Arg257*) variant [13]. However, for patient E the changes were so advanced that, although there was a macular atrophic area, the same phenotype was not distinguishable. 

For patient F, the ring of AF surrounding the atrophic change was different in its extent compared to the rest of this cohort. However, a similar phenotype was described in *CERKL* IRD before in a patient compound heterozygous for c.375C > G, and c.193G > T in Avela et al.’s Finnish *CERKL* cohort (patient 4 in Avela et al.) [10]. 

As described in other reports, peripheral involvement was variable, ranging from mild pigment clumping to severe widespread atrophy, as seen in patient E. Two of our cohort (B and F) had no visible peripheral changes at all. 

Patients D, E and F also possess variants in other known IRD genes (Table S2), but none of these variants were considered to be disease-causing, either because they were seen in a heterozygous state in genes causing autosomal recessive disease or because the phenotype previously described for the gene was not consistent. For example, Patient D was heterozygous for a *PDE6A* variant (c.769C > T p.(Arg257*)) of known pathogenicity and patient E was heterozygous for an I*MPG2* variant (c.789C > G p.(Ser263Arg)) of uncertain pathogenicity, but in both cases a second variant was not found (Table S2). Additionally, segregation studies for patient E identified the same *CERKL* variant (p.(Met349Valfs*20)) in the heterozygous state in his unaffected father. His unaffected mother is deceased and so was unavailable for analysis. Patient F was heterozygous for variants in *USH2A* and *NRL* (Table S2). The *USH2A*, c.3812-3_3837dup p.(Met1280*) is of presumed pathogenicity, however, the phenotype is not typical of an *USH2A*-related retinopathy and patient F is not deaf (although not all patients with *USH2A* variants have hearing impairment). The *NRL* c.11C > T p(Pro4Leu) variant is of uncertain pathogenicity and *NRL-*associated RP recessive or dominant is rare, and usually associated with a severe early-onset phenotype, which is not present in patient F, who was 40 years old at the age of diagnosis [55]. In addition, although only the segregation of the *CERKL* variants were investigated, her affected brother possesses the same two *CERKL* variants, whereas her unaffected brother has neither. 

Herein, we describe a cohort of six ethnically diverse patients, including white Caucasian, Indian, Pakistani, and Kashmiri origins. Previous reports in Finnish [10], Yemeni Jewish [11] and Spanish populations [13] reported the *CERKL* variants (c.375C > G, p.(Cys125Trp); c.238 + 1G > A and c.847C > T, p.(Arg283*) respectively) as a significant cause of arRP due to founder mutations. Three of our cohort, which did not include any patients from these populations, did have the c.847C > T variant found in the Spanish population. However, in other populations, *CERKL* variants are a rare cause of arRP, so there are limited phenotypic data in the literature [10,43]. The *CERKL* phenotype reported was variable in severity, degree of field loss and presence or lack of abnormal pigmentation. However there appeared to be a typical phenotype of early macular involvement with progression over time with concurrent varying degrees of progressive photoreceptor degeneration. The macular atrophy, seen particularly clearly on autofluorescence imaging, may be associated with a preserved small residual island of tissue [36]. As yet, there is no clear explanation for its persistence. It is also observed in some other IRDs, such as *ABCA4* retinopathies; Bax et al. made the observation that these patients were in their fifth decade and that foveal sparing is observed in patients with a low rate of progression [56]. The Finnish cohort described by Avela et al. demonstrates a wide range of severity, as also seen in our cohort. Recently, Yu et al. demonstrated progressive degeneration of rod and cone outer segments in a *Cerkl*-knockout zebrafish model, with rod degeneration preceding cone degeneration [57]. There was no clearly observed genotype phenotype correlation, either in our cohort or in the literature, in patients with pathogenic variants in *CERKL*. The variants described to date (Table 1) are distributed across the entire gene (Figure 1), and the exact function of *CERKL* remains unclear. Although it shows 50% similarity to the ceramide kinase (*CERK*) protein, which phosphorylates ceramide to ceramide 1-phosphate and encodes a potential diacylglycerol kinase (DAG) domain, there is no evidence of any kinase activity in *CERKL* [6]. Indeed, studies examining the transcriptional complexity of *CERKL* in the retina demonstrate that there are isoforms that do not contain the DAG domain [8,9]. There is some evidence for *CERKL* having a role in protecting retinal cells from injury caused by oxidative stress [9,58]. A recent study describing the generation of a mouse model using CRISPR-Cas9 editing to delete the *Cerkl* locus showed that total ablation of the locus was embryonically lethal [7]. The authors therefore generated a model where the knockout allele (*Cerkl^KO^*) was in *trans* with a knockdown allele (*Cerkl^KD^*). These animals showed defects in the photoreceptor outer segments but did not initially show any alteration in the electrophysiological response, although older mice did show some changes [7]. This model may prove to be useful in gaining some understanding of the function of *CERKL/Cerkl* in the mammalian retina.

To date, there are no therapeutic strategies to correct or treat the retinal degeneration caused by mutations in *CERKL.* However, gene therapy for *CERKL* using adeno-associated viral (AAV) vectors is a possibility due to a combination of the recessive nature of the disease and small coding sequence at 1599 base pairs, which is easily encodable by adeno-associated viral (AAV) vectors similar to Luxturna, which is approved for Leber’s Congenital Amaurosis (LCA) type II. One problem, however, is that *CERKL* may also have a vital role in the inner retina [4], but in this series we did not identify any significant inner retinal changes. Identifying the correct mRNA sequence will be essential in developing future gene therapy treatments, but *CERKL* has many known splice isoforms [8]. Hence, our observation of novel mutations in exons 2, 3, 6 and 8 confirms the likely critical role of these exons in the *CERKL* isoform required in photoreceptors.

## 5. Conclusions

In conclusion, *CERKL* mutations are an uncommon cause of arRP, but they are a significant cause of disease in populations with founder mutations [10,11,13]. The importance of seeking further information from family segregation studies is highlighted by the families described in this study. Identifying the correct genetic variant will be of significant importance when potential therapies become available. It is highly likely that, in some cases, revisiting the genotype, particularly in cases where more than one IRD gene variant is identified, will be required. This study adds to the phenotype spectrum of pathogenic variants in *CERKL* and highlights specific difficulties in diagnosis when more than one gene variant is present.

## Figures and Tables

**Table 1:** Variants identified in *CERKL* to date. Numbering is based on transcript NM_001030311 which contains 14 coding exons. Variants identified in this study are shaded blue.

Variant			Effect	References
c.156_157insT	Exon 1	GAG > TGA	p.(Glu53*)	[15]
c.193G > T	Exon 1	GAG > TAG	p.(Glu65*)	[10,16]
c.197_200dupGAGC	Exon 1	CTG > AGC	p.(Leu68Serfs*15)	[17,18]
c.238 + 1G > A	Intron 1		Splicing defect	[11,19]
Whole exon deletion	Exons 1 and 2		Loss of function	[20]
Whole exon deletion	Exon 2		Loss of function	[17]
c.316C > T	Exon 2	CGT > TGT	p.(Arg106Cys)	[10,16]
c.316C > A	Exon 2	CGT > AGT	p.(Arg106Ser)	[18,21] This report
c.356G > A	Exon 2	GGT > GAT	p.(Gly119Asp)	[22]
c.365T > G	Exon 2	CTC > CGC	p.(Leu122Arg)	[23]
c.375C > G	Exon 2	TGC > TGG	p.(Cys125Trp)	[10,16,24]
c.398T > C	Exon 2	CTA > CCA	p.(Leu133Pro)	[25]
c.420delT	Exon 2	CTT > CTA	p.(Leu140Leufs*2)	[26]
c.450_451delAT	Exon 2	ATA > ATG	p.(Ile150Metfs*3)	[27]
c.451T > G	Exon 2	TGG > GGG	p.(Trp151Gly)	[28]
c.481 + 2T > G	Intron 2		Splicing defect	[29]
c.566_569delinsGTG	Exon 3		p.(Lys189Serfs*5)	This report
c.598A > T	Exon 3	AAA > TAA	p.(Lys200*)	[26,30]
c.613 + 4_613 + 5delAG	Intron 3		Splicing defect	[18]
c.613 + 5_613del8	Intron 3		Splicing defect	[31]
c.664C > T	Exon 4	CAG > TAG	p.(Gln222*)	[32]
c.812T > C	Exon 6	CTG > CCG	p.(Leu271Pro)	[30,33]
c.836delT	Exon 6	ATG > AGG	p.(Met279Argfs*6)	[15,28]
c.847C > T	Exon 6	CGA > TGA	p.(Arg283*)	[4,10,13,16,17,18,22,24,26,30,32,34,35,36,37,38,39,40,41,42,43] This report
c.858delT	Exon 6	ACT > ACC	p.(Thr260Thrfs*10)	[26]
c.890T > C	Exon 6	ATA > ACA	p.(Ile297Thr)	[19,30]
c.899-1G > A	Intron 6		Splicing defect	[43]
c.999C > A	Exon 8	TGC > TGA	p.(Cys333*)	[44]
c.1045_1046delAT	Exon 8	ATG > GTT	p.(Met349Valfs*20)	[16,43,45] This report
c.1090C > T	Exon 8	CGA > TGA	p.(Arg364*)	[22,46] This report
c.1151 + 3_1151 + 6delAAGT	Intron 8		Splicing defect	[12,18]
c.1164_1165delTG	Exon 9	TGT > TGA	p.(Cys388*)	[27,36]
c.1270C > T	Exon 11	CAG > TAG	p.(Gln424*)	[29]
c.1303C > T	Exon11	CGA > TGA	p.(Arg435*)	[39]
c.1347 − 3C > G	Intron 11		Splicing defect	[18]
c.1381C > T	Exon 12	CGA > TGA	p.(Arg461*)	[38]
c.1393C > T	Exon 12	CGG > TGG	p.(Arg465Trp)	This report
c.1467_1470dupTTAC	Exon 13	ACT > TTA	p.(Thr491Leufs*4)	[47]
c.1482delA	Exon 13	GAA > GAG	p.(Glu494Glufs*1)	[28,48]
c.1617-16_1630inv30	Partial intron 13/exon 14			This report
c.1651A > T	Exon 14	AGC > TGC	p.(Ser551Cys)	[17]
c.1693_1696dupCTTT	Exon 14	TAT > TCT	p.(Tyr548Serfs*19)	[25]

**Table 2:** Clinical Information.

Patient	Gender	Ethnicity	Symptoms at Presentation	Age at First Presentation	Age at Last Visit	Lens Status		Visual Acuity (logMAR) at Last Visit		Visual Fields (Horizontal Loss)		Fundoscopy	Variant
						OD	OS	OD	OS	OD	OS		
A	M	White Caucasian (Irish)	↓ colour vision nyctalopia	25	35	N	N	6/24	HM	NP	NP	RP phenotype with widespread pigment, disc pallor, attenuated vessels and central atrophy sparing the fovea.	p.(Arg283*) Hom
B	F	Indian	↓ central vision slow dark adaptation	45	60	N	N	2/60	6/36	Central loss extending to 10–30°	Central loss extending to 20–30°	No pigment, central atrophy with foveal sparing.	p.(Arg283*) p.(Arg364*)
C	M	White Caucasian	↓ central vision, photopsia, nyctalopia and field loss	20	56	Pseudophakic	Pseudophakic	NPL	NPL	NP	NP	RP phenotype with widespread pigment, disc pallor, attenuated vessels and central atrophy with strip of foveal sparing.	p.(Arg283*) p.(Lys189Serfs*5)
D	F	Pakistani	Photosensitivity ↓ colour vision ↓ central vision field loss, photopsia, nyctalopia	25	30	N	N	6/18	6/12	<10°	<10°	Sparse pigmentation, disc pallor, attenuated vessels; distinct nummular RPE depigmentation in the mid-periphery with chorioretinal atrophic patches temporally.	c.1617-16_1630inv30 Hom
E	M	Indian	↓ central vision, nyctalopia	7	14	PSCLO	PSCLO	POL	POL	NP	NP	Dense pigmentation throughout the fundus, disc pallor, significantly attenuated vessels; widespread atrophy throughout fundus.	p.(Met349Valfs*20) Hom
F	F	Kashmiri	↓ central vision	40	57	N	N	6/60	6/60	NP	NP	No pigment, central atrophy, normal colour discs.	p.(Arg465Trp) p.(Arg106Ser)

Abbreviations: ↓ = reduced; AF=autofluorescence; HM = hand movements; N = normal; NA = not available; NP = not performed; NPL = no perception of light; POL = perception of light; PSCLO = posterior subcapsular lens opacities.

**Table 3:** Phenotypic features observed on autofluorescence and optical coherence tomography imaging in patients A–F.

Patient	Autofluorescence	CMT OD (μm)	CMT OS (μm)	OCT
A	Well demarcated dense loss of signal centrally, less dense atrophy external to arcades; increased focal AF signal at foveae, surrounded by atrophy within maculae.	266	237	Significant loss of ellipsoid zone in central retina with foveal sparing at site of increased AF signal; thin retinas, epiretinal changes.
B	Atrophy primarily confined to macular region with concentric patchy loss of signal and increased signal extending to arcades; foveal preservation.	229	266	Significant loss of outer retinal layers in macular region, with central foveal preservation in remnant corresponding to AF increased signal.
C	Loss of signal consistent with central atrophy with a strip of minimal central sparing. There were also peripheral areas of loss-of-signal consistent with the chorioretinal atrophic patches.	210	193	Loss of outer retina bilaterally.
D	Increased signal in the maculae surrounding the foveal atrophy, with patchy, punctate loss extending from and including the arcades.	176	183	Focal foveal disruption of ellipsoid layer and loss of the outer retinal layers in both eyes.
E	Complete loss of signal consistent with coalescing patches of atrophy throughout the fundi, including both maculae.	279	285	Loss of outer retina bilaterally with thickened inner retina.
F	AF imaging showed significant central loss of signal denoting atrophy with a thick band of increased AF extending to the disc and around the circumference of the atrophy.	126	131	Significant outer retinal loss in both eyes.

## Supplementary Materials

The following are available online at https://www.mdpi.com/2073-4425/11/12/1497/s1, Figure S1: Goldmann visual fields showing two timepoints in patients B and D; Figure S2: Fundus fluorescein angiography; Table S1: The thickness of the ganglion cell layer–inner plexiform layer in the nasal and temporal retina; Table S2: Variants identified in IRD genes other than *CERKL*.
