# Supplementary material for: Genetic and Clinical Findings in an Ethnically Diverse Cohort with Retinitis Pigmentosa Associated with Pathogenic Variants in CERKL
Source: Genes (Basel). 2020 Dec 12;11(12):1497. doi: 10.3390/genes11121497 (PMC7763961; doi:10.3390/genes11121497)
Supplement: Supplementary file 1 [file genes-11-01497-s001.pdf]

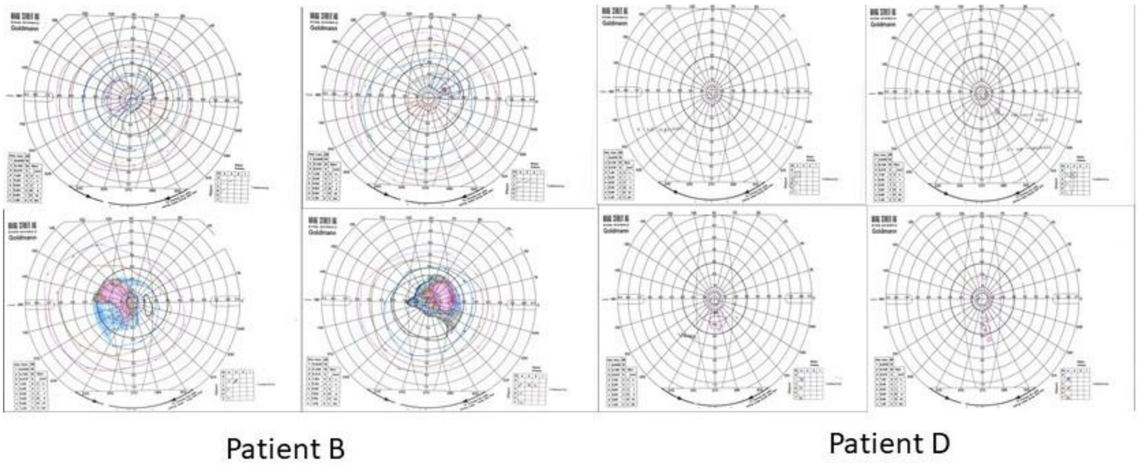

Figure 1. Goldmann visual fields showing two timepoints in patients B and D. Patient B the Goldmann visual fields show an increase in the central scotoma bilaterally over a 5 year time period. Patient D has severely constricted visual fields which are similar at a 2 year follow up.

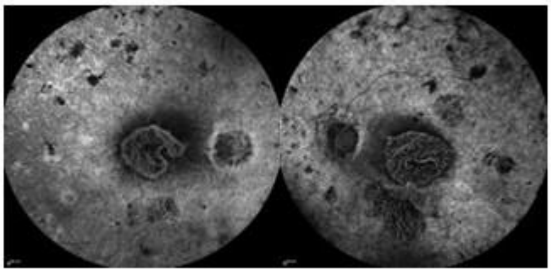

Figure 2. Fundus fluorescein angiography demonstrating central and inferior retinal patches of total retinal loss in patient C**.**

Table 1. Variants identified in IRD genes other than *CERKL***.**

Patient	Panel used	Gene	Variant		State	Report
**C**	55	*CACNA1F*	c.4343C>T	p.(Thr1448Ile)	Het	Uncertain
D	55	*PDE6A*	c.769C>T	p.(Arg257*)	Het	Pathogenic
E	111	*IMPG2*	c.789C>G	p.(Ser263Arg)	Het	Uncertain
G	111	*USH2A*	c.3812-3_3837dup	p.(Met1280*)	Het	Pathogenic
		NRL	c.11c>T	p.(Pro4Leu)	Het	Uncertain
